# Kinsenoside Ameliorates Oxidative Stress-Induced RPE Cell Apoptosis and Inhibits Angiogenesis via Erk/p38/NF-κB/VEGF Signaling

**DOI:** 10.3389/fphar.2018.00240

**Published:** 2018-03-20

**Authors:** Xu Luo, Shengjie Gu, Yujiao Zhang, Jianhong Zhang

**Affiliations:** Department of Ophthalmology, Shanghai Fourth People’s Hospital, Shanghai, China

**Keywords:** age-related macular degeneration, oxidative stress, kinsenoside, retinal pigment epithelium, vascular endothelial growth factor, NF-κB, MAPK

## Abstract

The pathological superoxidative condition that retinal pigment epithelium (RPE) cells experience contributed to the advancement of age-related macular degeneration (AMD), which was accompanied by significant neovascularization. Therefore, the discovery of novel pharmacological candidates to ameliorate oxidative damage (H_2_O_2_) against RPE cells and inhibit the following angiogenesis simultaneously is urgently needed. Herein, we found that kinsenoside (Kin), an active component derived from *Anoectochilus roxburghii*, was able to protect RPE cells effectively and attenuate subsequent angiogenesis. In this study, H_2_O_2_-induced oxidative injury reduced RPE cell viability and increased cell apoptosis, which was significantly rescued by the treatment with Kin. Compared with H_2_O_2_ alone, Kin decreased the levels of Bax and increased the production of Bcl-2 in RPE cells. H_2_O_2_-stimulated VEGF up-regulation was inhibited by Kin treatment. Human umbilical vein endothelial cell (HUVEC) neovascularization induced by conditioned medium (CM) from H_2_O_2_-stimulated RPE cells was attenuated by treatment with Kin, VEGF antagonist, NF-κB, Erk-MAPK, and p38-MAPK inhibitors. Additionally, H_2_O_2_-activated phosphorylated expression of IκBα, p65, Erk, and p38 in RPE cells was inhibited by treatment with Kin. Taken together, Kin protected RPE from apoptosis against oxidative stress while simultaneously decreasing apoptosis-related neovascularization. This could be ascribed to the inhibition of Erk/p38/NF-κB signaling by Kin that contributed to the resulting decreased VEGF expression in H_2_O_2_-treated RPE cells.

## Introduction

Age-related macular degeneration is the leading cause of irreversible visual disability among the elderly population, characterized by the accumulation of drusen between Bruch’s membrane in macular and RPE in the early stage and interconvertible “wet” and “dry” patterns in the late stage (Jager et al., 2008). Herein, CNV featuring excrescent blood vessel growth from choroidal membrane marks the progress of wet AMD, while the apoptotic loss of RPE cells indicates the advanced form of dry AMD (de Jong, 2006; Ehrenberg and Benny, 2017). Epidemiological studies have shown that numerous genetic and environmental factors contribute to the threshold of AMD, for instance, age, gender, race, smoking, and diet, among which age and smoking have been proven to be the most closely linked factors (Coleman et al., 2008). With regard to the underlying molecular mechanisms, it is acknowledged that oxidative stress, VEGF-induced angiogenesis and inflammation are the major pathogenic causes of AMD (Wei et al., 2001; Nowak et al., 2003; King et al., 2005).

The severity of oxidative stress in AMD is dependent on its significant induction of apoptosis in RPE cells (Cai et al., 2000). RPE cells, serving as a one-cell layer in the eye between the photoreceptors and the vascular choroid, play vital roles in providing nutritional and structural support for the retina. By protecting the retina from excess light and maintaining photoreceptor survival, RPE cells are capable of modulating sub-retinal ion balance and promoting the clearance of photoreceptor outer segments (POSs) (Kevany and Palczewski, 2010). Unfortunately, they do not renew after differentiation (Ebrahimi et al., 2017), inevitably resulting in RPE apoptosis caused by long-term oxidative damage. In severe AMD cases, these apoptotic RPE cells release VEGF, the most significant pro-angiogenic factor, resulting in the activation of CNV and the further development of wet AMD (Dinc et al., 2017). Therefore, it is imperative to inhibit RPE cell apoptosis for abrogating subsequent angiogenesis in AMD. However, anti-AMD remedies commonly used in the clinic currently such as anti-VEGF injection, laser photocoagulation and photodynamic therapy possess several drawbacks since they target RPE apoptosis and CNV separately while ignoring the close crosstalk between these distinct bio-steps. Hence, there is an urgent need for the development of novel anti-AMD therapeutic strategies that simultaneously protect RPE cells and inhibit the following neovascularization.

Kinsenoside, an active drug component derived from the traditional Chinese medicine herb, *Anoectochilus roxburghii*, has been shown to exert comprehensive pharmacologic effects in treating gouty arthritis, hyperliposis, osteoporosis, and autoimmune hepatitis (Du et al., 2001; Hsiao et al., 2013; Han et al., 2016; Xiang et al., 2016). By targeting stimulus-induced macrophages, Kin was able to protect endothelial cells from inflammation-triggered apoptosis directly and indirectly, showing significant vascular protective capabilities of Kin under damaging conditions (Liu et al., 2013; Han et al., 2016). Despite the extensive applications in pre-clinical translational research of Kin, the potential effects of Kin in targeting RPE cells and treating subsequent CNV remain largely unexplored. More importantly, the combined treatment against RPE damage and neovascularization under AMD conditions should be addressed simultaneously. Therefore, we intended to first utilize superoxidative stress (H_2_O_2_) to mimic the oxidative conditions of AMD and then investigate whether Kin could reverse H_2_O_2_-induced RPE apoptosis and inhibit subsequent CNV with the clarified molecular mechanisms. By taking advantage of Kin, we aimed for this study to provide novel insights into AMD treatment and to pave a solid foundation for the future translational practice of Kin clinically.

## Materials and Methods

### Cells, Media, and Reagents

Human RPE cell line (ARPE-19) and HUVECs were cultured in α-MEM medium (HyClone, Logan, UT, United States) supplemented with 10% fetal bovine serum (FBS) (HyClone, Logan, UT, United States) and 1% penicillin/streptomycin (Gibco, Invitrogen, Carlsbad, CA, United States) in humidified incubators with 5% CO_2_ at 37°C. Kin was obtained from Shifeng Biological Technology Company (Shanghai, China). H_2_O_2_ solution was obtained from Beyotime Biotechnology (Shanghai, China) and kept at 4°C while protecting from light. The CM from RPE cells contained serum-free medium and 5% bovine serum albumin (Sigma-Aldrich, St. Louis, MO, United States) (Han et al., 2016). All of the primary and secondary antibodies were obtained from Cell Signaling Technology (Danvers, MA, United States) unless otherwise specified.

### Cell Viability

Cell viability of RPE cells was determined with the CCK-8. Cells were treated with various concentrations of H_2_O_2_ and Kin for 24 h. The optical density at 450 nm (OD_450_) was then measured with a microplate reader after incubation with mixed CCK-8 solution at 37°C for 2 h, according to manufacturer’s instructions (Qiao et al., 2016). Each experiment was repeated at least three times.

### Cell Apoptosis

Cell apoptosis of RPE cells was determined by flow cytometry by Annexin V/propidium iodide (PI) double immunofluorescent staining, according to manufacturer’s instructions (Qiao et al., 2017). RPE cells were treated with H_2_O_2_ and varying dosages of Kin for 24 h. Cell apoptosis rates (%) were then analyzed by the Annexin V/PI Staining Kit (BD Biosciences, San Jose, CA, United States) by adding the upper right (FITC^+^/PI^+^) and lower right (FITC^+^/PI^-^) cell events. Each experiment was repeated at least three times.

### Western Blotting

Retinal pigment epithelium cells were treated with H_2_O_2_ and varying dosages of Kin for 24 h. Proteins were then extracted with RIPA buffer (Beyotime Biotechnology, Shanghai, China) supplemented with a cocktail of protease inhibitors and phosphatase inhibitors (Thermo Fisher Scientific, Hanover Park, IL, United States). Protein concentrations were confirmed with the BCA protein kit (Thermo Fisher Scientific, Hanover Park, IL, United States), and 30 μg of protein was separated by SDS-PAGE and transferred to a nitrocellulose (NC) membrane to detect protein expression. Western blotting bands were acquired with an Odyssey Infrared Imaging System (LI-COR Biosciences, Lincoln, NE, United States), and band intensities were quantified according to previous reports (Song et al., 2017). Each experiment was repeated at least three times.

### VEGF Determination

Supernatants from RPE cells treated with H_2_O_2_, and varying dosages of Kin for 24 h were collected to determine the concentrations of VEGF after stimulation via an ELISA kit (R&D System, Minneapolis, MN, United States), according to the manufacturer’s guidelines. Each experiment was repeated at least three times.

### CLSM Observations of Neovascularization

Human umbilical vein endothelial cells (7 × 10^4^ cells/well) were suspended and seeded in matrigel (BD Biosciences, San Jose, CA, United States)-coated (100 μL/well) 48-well plates. The CM from RPE cells treated with H_2_O_2_ and Kin for 24 h was collected and applied to HUVECs at 37°C. Also, the CM was changed at least once to remove the H_2_O_2_ and Kin from the media in order to collect only the CM. After 8 h, HUVECs were stained with calcein-AM (2 μmol/L) (BD Biosciences, San Jose, CA, United States), and tube formation was observed under CLSM (Sun et al., 2017). Each experiment was repeated at least three times.

### NF-κB/MAPK/VEGF Inhibitors Treatments

Human umbilical vein endothelial cells (7 × 10^4^ cells/well) were suspended and seeded in matrigel-coated (100 μL/well) 48-well plates. NF-κB inhibitor BAY 11-7082 (500 nmol/L), Erk-MAPK inhibitor SCH772984 (250 nmol/L), p38-MAPK inhibitor SB 202190 (1 μmol/L), and VEGF antagonist sFlt-1 (2.5 μmol/L) (Berger et al., 2012; Pan et al., 2015; Gu et al., 2017; Tang et al., 2018), at non-toxic concentrations were added to RPE cells, from which CM was collected to treat HUVECs in order to unravel the possible anti-AMD mechanism of Kin. After 8 h, HUVECs were stained with calcein-AM (2 μmol/L), and tube formation was observed under confocal microscopy. Each experiment was repeated at least three times.

### Statistical Analysis

Analysis of variance (ANOVA) was used to evaluate the statistical significance of data acquired among varying groups, where *P* < 0.05 was considered significant. Data were assessed with SPSS 13.0 software (Statistical Package for the Social Sciences, Chicago, IL, United States) and presented as the means ± SD.

## Results

### Kin Protects H_2_O_2_-Induced RPE Cell Death

**FIGURE 1:**
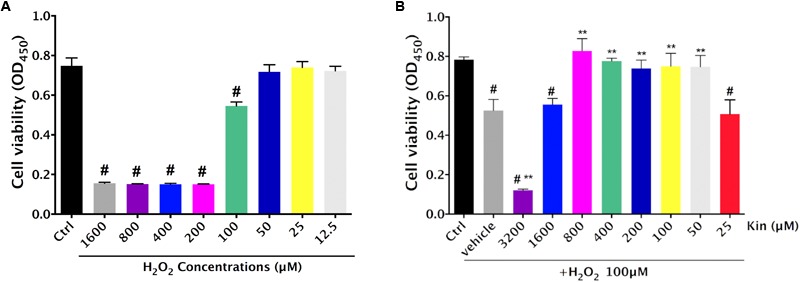
Cytotoxic effects of H_2_O_2_-induced oxidative stress and Kin in RPE cells. **(A)** Cell viability of RPE cells was assessed after various H_2_O_2_ treatments alone. **(B)** Cell viability of RPE cells was evaluated after combined treatments of 100 μM of H_2_O_2_ and different Kin concentrations. The data are presented as the means ± SD. ^#^*p* < 0.05 compared with Control group, ^∗∗^*p* < 0.05 compared with Vehicle group. All data were obtained from at least three independent experiments.

The RPE cell viability decreased significantly following oxidative administration (H_2_O_2_) in a dose-dependent manner (**Figure 1A**). In comparison with the Control group (0 μM H_2_O_2_-treated RPE group), RPE cells treated with 1,600, 800, 400, 200, and 100 μM of H_2_O_2_ showed a remarkable decrease in viability, while those treated with 50, 25, and 12.5 μM of H_2_O_2_ exhibited no significant difference. Hence, in order to induce significant H_2_O_2_-triggered RPE apoptosis for AMD modeling, 100 μM of H_2_O_2_ was deployed for further experiments.

Under the application of 100 μM of H_2_O_2_, 3,200 μM of Kin resulted in significant induction of cell death compared with the Vehicle group (100 μM of H_2_O_2_ treatment only), whereas 1,600 μM of Kin showed negligible difference (**Figure 1B**). Interestingly, 800, 400, 200, 100, and 50 μM of Kin increased RPE cell viability dramatically compared with the Vehicle, suggesting that Kin could protect RPE cells from H_2_O_2_-induced cell death. No cell revival was witnessed in the 25 μM of Kin group in comparison with the Vehicle group.

### Kin Attenuates H_2_O_2_-Induced RPE Cell Apoptosis

**FIGURE 2:**
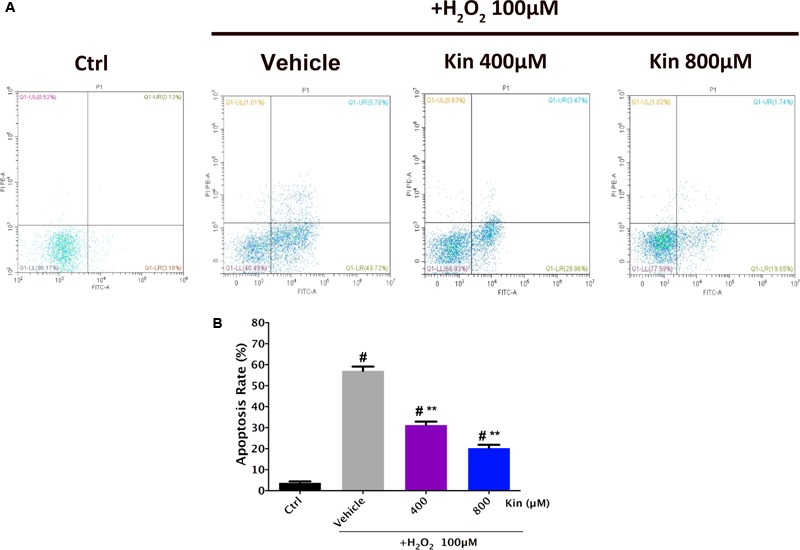
Apoptosis induction effects of H_2_O_2_-stimulated oxidative stress and Kin in RPE cells. **(A)** Cell apoptosis rates were analyzed after RPE cells were treated with combined 100 μM of H_2_O_2_ and 400 or 800 μM of Kin. **(B)** The additions of the upper right (FITC^+^/PI^+^) and lower right (FITC^+^/PI^-^) cell apoptosis rates were calculated. The data are presented as the means ± SD. ^#^*p* < 0.05 compared with Control group, ^∗∗^*p* < 0.05 compared with Vehicle group. All data were obtained from at least three independent experiments.

Since 800, 400, 200, 100, and 50 μM of Kin could protect RPE cell viability against H_2_O_2_-induced damage, 400 and 800 μM of Kin were employed for further cellular apoptosis analyses. Flow cytometry revealed that H_2_O_2_ stimulation was able to generate a sharp increase in the apoptotic RPE populations in the Vehicle group (57.09 ± 1.42%) compared with the Control group (3.76 ± 0.45%) (**Figures 2A,B**). In contrast, treatments with 400 and 800 μM of Kin resulted in decreased apoptosis rates, reaching 31.32 ± 1.11% and 20.3 ± 1.09%, respectively. The aforementioned data indicated that despite the well-proven effects of oxidative stress in inducing RPE viability decrease and apoptosis increase, Kin was shown for the first time to exhibit a significant RPE protective capability in rescuing cell viability and attenuating cell apoptosis, implying a potential application in future AMD treatment.

### Kin Inhibits RPE Apoptosis by Modulating Apoptosis-Related Proteins Bax/Bcl-2 Expression

**FIGURE 3:**
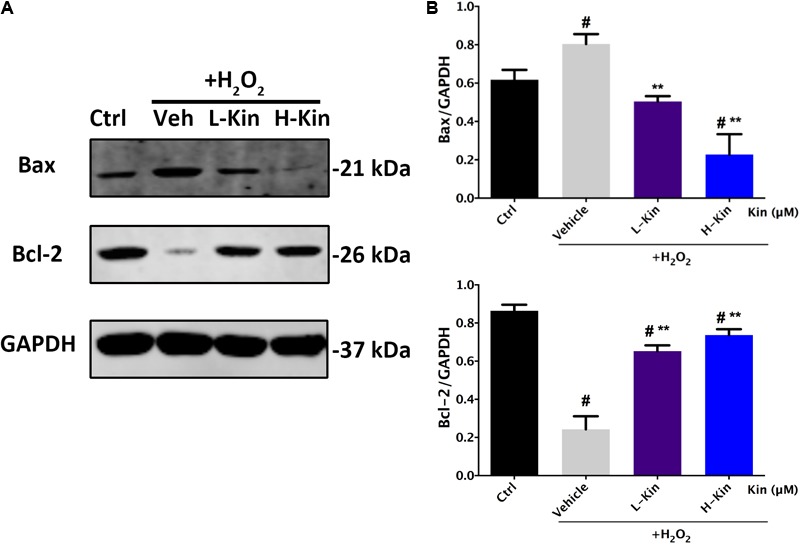
The expression of apoptosis-related proteins in RPE cells treated with H_2_O_2_ and Kin. **(A)** Kin treatments decreased H_2_O_2_-induced Bax expression and increased H_2_O_2_-attenuated Bcl-2 levels. **(B)** Protein levels of Bax and Bcl-2 were quantified by gray scale. The data are presented as the means ± SD. ^#^*p* < 0.05 compared with Control group, ^∗∗^*p* < 0.05 compared with Vehicle group. All data were obtained from at least three independent experiments.

Because the apoptosis-inhibiting ability was more evident than the viability-protecting ability of Kin, the underlying anti-apoptosis mechanism of Kin was selected for further investigation on the basis of the above results. **Figures 3A,B** illustrated that Bax (the pro-apoptotic protein) was up-regulated following H_2_O_2_ stimulation in the Vehicle group, while both low Kin (400 μM) and high Kin (800 μM) treatments decreased such tendencies. Furthermore, for Bcl-2 protein (the anti-apoptotic protein) expression, the Vehicle group showed a significant decrease, whereas both low and high Kin reversed this attenuation. These results indicated that the protective ability of Kin against H_2_O_2_-induced RPE apoptosis was partly attributed to the regulation of the Bax/Bcl-2 ratio, implicating the possible modulation of the mitochondrial-dependent cell death pathway (Wang et al., 2017) by Kin in H_2_O_2_-treated RPE cells.

### Kin Inhibits H_2_O_2_-Induced VEGF Release in Apoptotic RPE Cells

**FIGURE 4:**
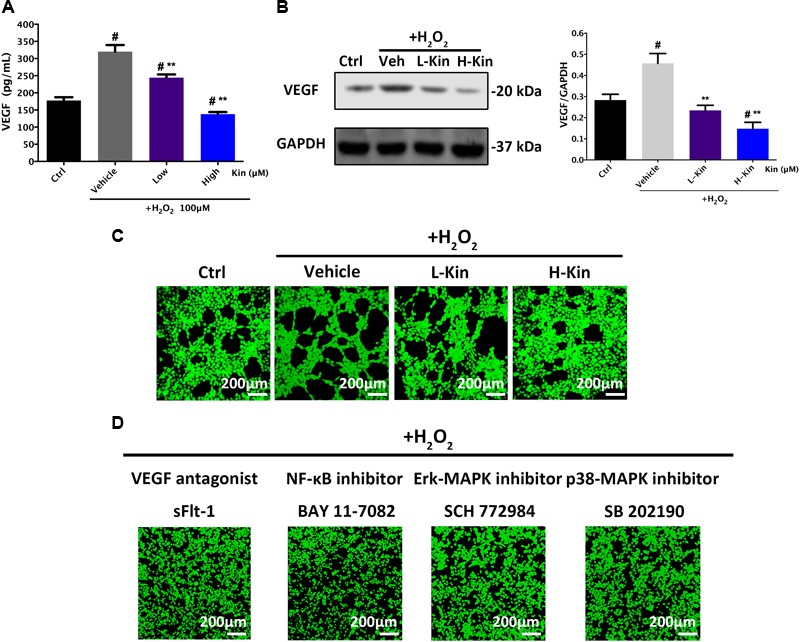
The expression of VEGF in RPE cells and subsequent apoptotic RPE-induced neovascularization in HUVECs. **(A)** Measurements of the secretion of VEGF from RPE cells treated with H_2_O_2_ and Kin. **(B)** Production of VEGF in RPE cells treated with H_2_O_2_ and Kin. Protein levels of VEGF were quantified by gray scale. **(C)** The neovascularization of HUVECs added with CM from H_2_O_2_ and Kin treated RPE cells. **(D)** The neovascularization of HUVECs added with CM from H_2_O_2_ and VEGF antagonist sFlt-1, NF-κB inhibitor BAY 11-7082, Erk-MAPK inhibitor SCH772984, plus p38-MAPK inhibitor SB 202190, treated RPE cells. The data are presented as the means ± SD. ^#^*p* < 0.05 compared with Control group, ^∗∗^*p* < 0.05 compared with Vehicle group. All data were obtained from at least three independent experiments.

It is well-known that H_2_O_2_ administration contributes to RPE cell apoptosis, leading to increased levels of VEGF expression from RPE cells that results in the undesirable CNV in AMD cases (Dinc et al., 2017). Herein, we showed that both low and high concentrations of Kin were able to attenuate VEGF secretion from H_2_O_2_-induced RPE cells in a dose-dependent manner (**Figure 4A**). These results were then confirmed by Western blotting (**Figure 4B**), which showed significant VEGF-inhibitory effects by Kin treatment.

### Kin Inhibits Apoptotic RPE-Stimulated Neovascularization

Furthermore, CM from H_2_O_2_-treated RPE cells resulted in significantly increased angiogenesis in comparison with the Control group, as evidenced by the formation of more and larger tubules in HUVECs stimulated with H_2_O_2_-treated CM (**Figure 4C**). Nevertheless, the low concentration of Kin inclined to inhibit neovascularization, showing less and smaller tube formation after CM treatment. Additionally, a high concentration of Kin reduced tube formation most significantly, exemplifying the fewest and smallest tubules in CM-treated HUVECs. These results indicated that Kin could not only protect RPE cells from oxidative stress-induced cell death but also inhibit apoptotic RPE cell-stimulated neovascularization, indicating the translational potential for AMD treatment clinically by targeting both RPE apoptosis and CNV, simultaneously.

### Effects of NF-κB/MAPK/VEGF Inhibitors on Apoptotic RPE-Induced Neovascularization

In addition, we used the VEGF antagonist, sFlt-1, at a non-toxic dosage to treat RPE cells with H_2_O_2_ and collected CM to further apply to HUVECs. Confocal imaging results showed that neovascularization was drastically inhibited, displaying scarce tube formation by HUVECs compared with the Vehicle group (**Figure 4D**), which signified the vital importance of VEGF during apoptotic RPE-induced neovascularization. Furthermore, H_2_O_2_-activated NF-κB and MAPK pathways that serve as upstream regulators of VEGF have been shown to control VEGF release (Berger et al., 2012; Klettner et al., 2013; Koinzer et al., 2015; Chong and Zheng, 2016; Swamynathan et al., 2017). Therefore, the NF-κB inhibitor, BAY 11-7082, Erk-MAPK inhibitor, SCH772984, and p38-MAPK inhibitor, SB 202190, at non-toxic concentrations were used to treat RPE cells for CM collections. As shown in **Figure 4D**, angiogenesis in HUVECs after CM-stimulations could barely be observed after NF-κB/MAPK inhibition, as exemplified by the irregular and random HUVEC distribution. No large and round tubule in HUVECs was seen after inhibitor administration, implicating the crucial regulative roles of activated NF-κB/MAPK signaling in modulating VEGF release in RPE cells.

### Inhibitory Effects Against p-IκBα/IκBα and p-p65/p65 in H_2_O_2_-Treated RPE Cells by Kin

**FIGURE 5:**
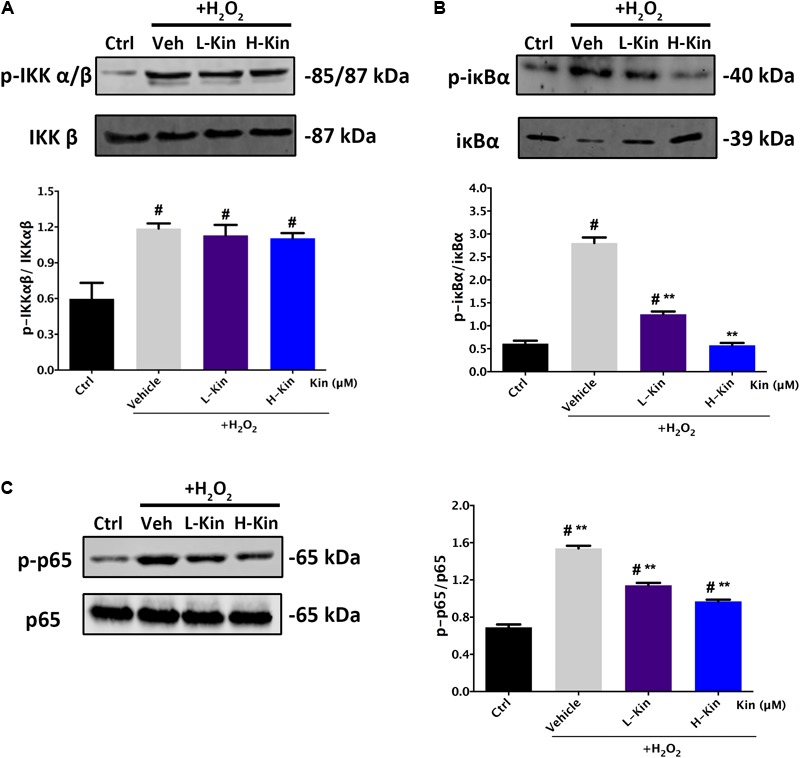
Effects of Kin on H_2_O_2_-induced NF-κB activation. Expression of NF-κB associated proteins **(A)** p-IKKαβ/IKKαβ, **(B)** p-IκBα/IκBα, and **(C)** p-p65/p65 in RPE cells treated with H_2_O_2_ and Kin. Protein levels were quantified by gray scale. The data are presented as the means ± SD. ^#^*p* < 0.05 compared with Control group, ^∗∗^*p* < 0.05 compared with Vehicle group. All data were obtained from at least three independent experiments.

It is shown in **Figure 5** that H_2_O_2_ activated NF-κB signaling by phosphorylating IKK α/β, IκBα, and p65 molecules. After Kin treatments, the levels of phosphorylated IκBα and p65 decreased significantly compared with the Vehicle group. However, the upstream protein IKK α/β was not affected by Kin administration. This suggested that Kin might target activated IκBα protein in RPE cells irrespective of upstream IKK α/β, thus inhibiting downstream p65 phosphorylation, which then contributed to the attenuation of the NF-κB pathway.

### Inhibitory Effects Against p-Erk/Erk and p-p38/p38 in H_2_O_2_-Treated RPE Cells by Kin

**FIGURE 6:**
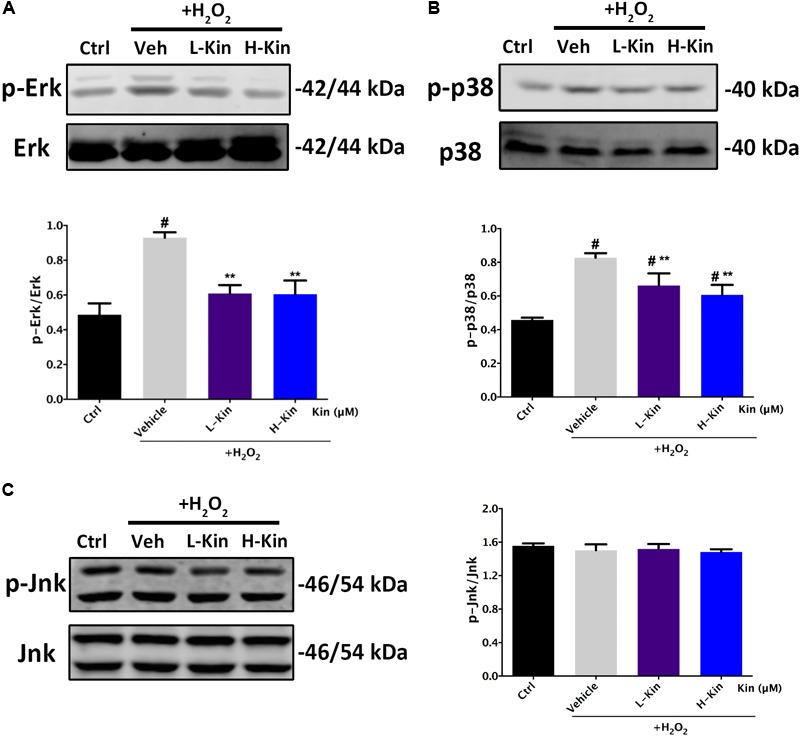
Effects of Kin on H_2_O_2_-induced MAPK activation. Expression of MAPK associated proteins **(A)** p-Erk/Erk, **(B)** p-p38/p38, and **(C)** p-Jnk/Jnk in RPE cells treated with H_2_O_2_ and Kin. Protein levels were quantified by grayscale. The data are presented as the means ± SD. ^#^*p* < 0.05 compared with Control group, ^∗∗^*p* < 0.05 compared with Vehicle group. All data were obtained from at least three independent experiments.

Next, we also assessed the phosphorylated levels of MAPK signaling in H_2_O_2_-treated RPE cells. As shown in **Figure 6**, both Erk and p38 molecules were activated by H_2_O_2_ administration. However, it was evident that Kin treatments reduced phosphorylated Erk and p38 levels regardless of Jnk, in comparison with the Vehicle group. This indicated that Kin was able to impede the activation of Erk and p38, resulting in the attenuation of the MAPK pathway.

**FIGURE 7:**
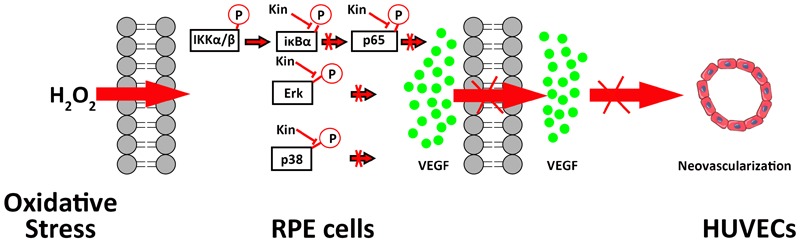
A schematic diagram of the possible mechanisms of Kin in attenuating VEGF-mediated apoptotic RPE cell-induced neovascularization. Kin could target H_2_O_2_-activated IκBα phosphorylation irrespective of upstream IKK α/β, thus inhibiting downstream p65 phosphorylation that contributed to the overall attenuation of the NF-κB pathway. Kin treatments also decreased phosphorylated Erk and p38 expression regardless of Jnk, illustrating that Kin could retard the H_2_O_2_-activated phosphorylation of Erk and p38, leading to the inhibition of the MAPK pathway. These altogether resulted in the decreased expression of VEGF in H_2_O_2_-treated RPE cells, contributing to the inhibited neovascularization in HUVECs.

On the basis of the above mechanistic analyses, it was illustrated that by targeting both NF-κB and MAPK signaling, Kin was capable of inhibiting H_2_O_2_-induced activation, thus down-regulating NF-κB/MAPK and downstream VEGF expression by RPE cells, contributing to the inhibited neovascularization by HUVECs (**Figure 7**).

## Discussion

The oxidative stress-induced RPE cell damage and the following CNV are two closely interlinked steps in the development of severe AMD, during which VEGF plays a core role (Kannan et al., 2006). Therefore, it is of great significance to control VEGF secretion from RPE cells against oxidative stress in the treatment of advanced AMD and CNV. Dinc et al. (2017) showed that H_2_O_2_-induced VEGF production by RPE cells could be decreased significantly by treatments of caffeic acid phenethyl ester (CAPE) alone and in combination with bevacizumab. In this study, aimed at solving oxidative stress-stimulated RPE apoptosis and neovascularization simultaneously, we found that Kin could not only attenuate RPE cell apoptosis but could also inhibit apoptosis-associated angiogenesis via down-regulating VEGF expression by RPE cells. To the best of our knowledge, this is the first study unraveling the therapeutic effects of Kin against eye diseases, showing its potential to achieve efficient protective effects for other hyperoxidative ocular diseases, such as diabetic retinopathy and proliferative vitreoretinopathy (PVR), extending the application of Kin.

Although there has been no research focusing on the use of Kin in treating eye diseases, ginsenoside, which was derived from glycosides, has been found to protect RPE cells from cobalt chloride (CoCl_2_) and hypoxic conditions (Li et al., 2013). This supports our experimental results from another angle. When evaluating the protective effects of Kin against H_2_O_2_-induced inhibition of RPE proliferation, we observed that despite the significant growth inhibition by 100 μM of H_2_O_2_ alone, medium concentrations of Kin were able to revive the RPE cells, as exemplified by the increased cell viability after Kin treatments. In contrast, high concentrations of Kin failed to protect RPE cells, possibly due to its cytotoxicity. This indicated that dosage-effect should be strictly taken into consideration provided that Kin would be applied in clinical practice. Additionally, a low concentration of Kin hardly influenced RPE injury, implying that the drug was diluted so greatly that no pharmacologic effect was displayed.

Since the expression of VEGF was intimately linked with the apoptosis state of RPE cells, which could be regulated by hyperoxidants, high glucose levels and cyclic stretch (De Cillà et al., 2017; Oh et al., 2017; Wu et al., 2017), the beneficial effects of Kin against oxidative apoptosis were further analyzed. We found that Kin drastically reduced apoptosis of RPE cells compared with the H_2_O_2_-treated Vehicle group. Additionally, studies have explored crucial mechanisms modulating cell apoptosis, including mitochondrial dependent and mitochondria-independent pathways (Hao et al., 2017; Tang et al., 2017). During the mitochondrial-dependent cascade, mitochondria are able to express cytochrome c, which then enters the cytosol to form a functional complex with apaf-1 protein (Li et al., 1997) for apoptosis. Herein, Bax and Bcl-2 that are derived from the Bcl-2 family of proteins are shown to translocate from the cytosol to mitochondria for apoptosis modulation (Wei et al., 2001). Specifically, Bax is capable of facilitating cytochrome c release, while Bcl-2 is found to impede cytochrome c movement. As a result, serving as a pro-apoptosis and anti-apoptosis protein, respectively, Bax and Bcl-2 are known to regulate cell apoptosis by altering the Bax/Bcl-2 ratio. Hence, the expression of Bax and Bcl-2 proteins in RPE cells was assessed after Kin and H_2_O_2_ treatments. The decreased Bax production plus the increased Bcl-2 level, in comparison with the Vehicle group, indicated the fact that Kin repressed the mitochondrial-dependent apoptosis pathway in RPE cells after oxidative stimulation. More importantly, by influencing the expression of Bax and Bcl-2 to impact RPE apoptosis, the following VEGF expression was decreased significantly from Kin-treated RPE cells under H_2_O_2_, as exemplified by the results obtained from ELISA and Western blotting. These implicated the possible effective treatment in attenuating neovascularization by Kin during wet AMD remedies.

On the basis of this, we collected CM from Kin- and H_2_O_2_-stimulated RPE cells to further administer to HUVECs, showing that Kin treatments could attenuate apoptotic RPE-induced angiogenesis effectively under a hyperoxidative bio-state. In addition, this tendency was akin to the treatment of the VEGF inhibitor, sFlt-1, signifying the potential similar effects between Kin with sFlt-1 by inhibiting VEGF expression from RPE cells to abrogate subsequent angiogenesis. This propelled us to explore the underlying VEGF-regulative mechanisms by Kin treatments in-depth. Klettner et al. (2013) showed that the activation of NF-κB and p38-MAPK pathways are involved in the up-regulated VEGF levels of H_2_O_2_-stimulated RPE cells. Moreover, phosphatidylinositol 3-kinase (PI3K), Jnk-MAPK and Erk-MAPK signaling (Bian et al., 2007; Koinzer et al., 2015) were also discovered to result in the increased production of VEGF for pro-neovascularization of RPE cells. Therefore, we used the NF-κB inhibitor, BAY 11-7082, Erk-MAPK inhibitor, SCH772984 and p38-MAPK inhibitor, SB 202190, at non-toxic concentrations and found that by inhibiting relevant pathways in RPE cells, the CM-induced angiogenesis was inhibited as well, implying the potentially similar mechanisms of Kin in VEGF regulation. This was confirmed with further Western blotting results, which showed that Kin could target H_2_O_2_-activated IκBα phosphorylation irrespective of upstream IKK α/β and, thus, inhibited downstream p65 phosphorylation that contributed to the overall attenuation of the NF-κB pathway. In addition, it was apparent that Kin treatments also decreased phosphorylated Erk and p38 expression regardless of Jnk, illustrating that Kin could also retard the H_2_O_2_-activated phosphorylation of Erk and p38, leading to the inhibition of the MAPK pathway. These altogether resulted in the decreased expression of VEGF in H_2_O_2_-treated RPE cells by Kin together with NF-κB pathway inhibition.

## Conclusion

Kinsenoside protected RPE from apoptosis against oxidative stress while simultaneously decreasing apoptosis-related neovascularization. This could be ascribed to the inhibition of Erk/p38/NF-κB signaling by Kin that contributed to the resulting decreased VEGF expression in H_2_O_2_-treated RPE cells, leading to the attenuated apoptotic RPE-induced neovascularization by HUVECs. Since our study is the first to apply Kin in the remedies of eye diseases, there are possibilities of Kin treatment to achieve effective therapeutic effects for other hyperoxidative ocular diseases, such as diabetic retinopathy and PVR, extending the application of Kin. However, further AMD animal experiments are required for the comprehensive evaluation of Kin treatments *in vivo*.

## Author Contributions

XL and JZ designed the experiments. XL conducted the experiments, analyzed the data, and wrote the manuscript. SG and YZ analyzed the data and reviewed the manuscript.

## Conflict of Interest Statement

The authors declare that the research was conducted in the absence of any commercial or financial relationships that could be construed as a potential conflict of interest.

## Abbreviations

AMD: age-related macular degeneration
Bax: Bcl-2 associated X protein
Bcl-2: B cell lymphoma-2
CCK-8: cell counting kit-8
CLSM: confocal laser scanning microscopy
CM: conditioned medium
CNV: choroidal neovascularization
ELISA: enzyme-linked immunosorbent assay
Erk: extracellular-regulated protein kinases
HUVECs: human umbilical vein endothelial cells
IκBα: inhibitor kappa B α
Jnk: c-Jun N-terminal kinase
Kin: kinsenoside
MAPK: mitogen-activated protein kinase
NF-κB: nuclear factor kappa B
RPE: retinal pigment epithelium
VEGF: vascular endothelial growth factor
